# Ecological rules for the assembly of microbiome communities

**DOI:** 10.1371/journal.pbio.3001116

**Published:** 2021-02-19

**Authors:** Katharine Z. Coyte, Chitong Rao, Seth Rakoff-Nahoum, Kevin R. Foster

**Affiliations:** 1 Division of Evolution and Genomic Sciences, Faculty of Biology, Medicine and Health, University of Manchester, Manchester, United Kingdom; 2 Department of Zoology, University of Oxford, Oxford, United Kingdom; 3 Department of Biochemistry, University of Oxford, Oxford, United Kingdom; 4 Division of Infectious Diseases, Division of Gastroenterology, Department of Pediatrics, Boston Children’s Hospital, Boston, Massachusetts, United States of America; 5 Department of Microbiology, Harvard Medical School, Boston, Massachusetts, United States of America; Instituto Gulbenkian de Ciencia, PORTUGAL

## Abstract

Humans and many other hosts establish a diverse community of beneficial microbes anew each generation. The order and identity of incoming symbionts is critical for health, but what determines the success of the assembly process remains poorly understood. Here we develop ecological theory to identify factors important for microbial community assembly. Our method maps out all feasible pathways for the assembly of a given microbiome—with analogies to the mutational maps underlying fitness landscapes in evolutionary biology. Building these “assembly maps” reveals a tradeoff at the heart of the assembly process. Ecological dependencies between members of the microbiota make assembly predictable—and can provide metabolic benefits to the host—but these dependencies may also create barriers to assembly. This effect occurs because interdependent species can fail to establish when each relies on the other to colonize first. We support our predictions with published data from the assembly of the preterm infant microbiota, where we find that ecological dependence is associated with a predictable order of arrival. Our models also suggest that hosts can overcome barriers to assembly via mechanisms that either promote the uptake of multiple symbiont species in one step or feed early colonizers. This predicted importance of host feeding is supported by published data on the impacts of breast milk in the assembly of the human microbiome. We conclude that both microbe to microbe and host to microbe interactions are important for the trajectory of microbiome assembly.

## Abstract 2

Humans and many other hosts establish a diverse community of beneficial microbes anew each generation, but what determines the success of the assembly process remains poorly understood. This study develops ecological theory that reveals the rules underlying the assembly of such host-associated microbiota.

## Introduction

Many multicellular organisms harbor dense microbial communities that are vital for host health; providing nutrients, protecting from pathogens, and promoting immune system development [1–4]. However, most hosts are not born with these diverse communities. Instead, their microbiomes gradually assemble after birth, progressing over time from a state of low diversity to form richer multispecies communities [5–8]. In many hosts, this developmental process is remarkably predictable, with the same characteristic taxa colonizing the gut at different points during development in most individuals [5–9]. And, crucially, the ordered nature of this acquisition of a diverse microbiome community is often considered critical for health. In corals, for example, symbionts that are beneficial to adults can be detrimental if acquired too early in development [10]. Meanwhile, in humans, failure to establish a stable and diverse microbiome is associated with numerous pathologies, including necrotizing enterocolitis, a devastating disease that causes significant morbidity and mortality in premature infants [11,12]. However, despite this importance, we still understand little about what causes a given microbiome community to assemble and persist.

While there is a large and rapidly growing body of empirical data on host-associated microbiomes [13], it remains challenging to disentangle the drivers of microbiome assembly, because so many species and processes can be potentially conflated. However, there is a long history in ecology—and many other areas, from weather prediction to neurobiology—of developing complementary theory to understand such complex systems. The great power of these methods is the ability to model vast numbers of different scenarios and thereby identify general principles that can elude empirical work alone. In this tradition, here we develop a theoretical framework to investigate potential drivers of microbiome community assembly.

We extend ecological network theory to ask, for any given community, what are the possible pathways by which it might assemble from an uncolonized environment. Our work suggests that diverse microbiome communities can most robustly assemble when the constituent species interact with one another weakly and in a predominantly noncooperative manner. When hosts rely on strong metabolic interactions within their microbiome, therefore, they face a problem as these interactions can limit community assembly. However, our theory also suggests that when they do occur, strong positive interactions may play a key role in increasing the predictability of community assembly. We also identify interventions that hosts can perform to overcome barriers to assembly, via selecting for ecologically dependent species, or promoting their co-colonization with partner species. Our models provide a framework with which to predict how an array of host-mediated and microbial interactions may affect the assembly process in any host–microbe system, and we illustrate this by supporting our key results with recently published data.

## Results and discussion

### A framework to study community assembly

We model microbiome communities using a generalized Lotka–Volterra (gLV) model. This system of ordinary differential equations describes how the growth rate of each species, *X*_*i*_, is determined by its own intrinsic properties, *r*_*i*_, its interactions with conspecifics, *s*_*i*_, and its interactions with other microbial species, *a*_*ij*_ (Materials and methods, section 1). This approach assumes that the ability of a species to maintain a viable population comes either from its capacity for independent growth (positive *r*_*i*_), or via benefits that come from others (positive *a*_*ij*_, e.g., using the metabolic breakdown products of other species), or a combination of both. These positive terms are in turn balanced by the negative effects of within-species density-dependent population regulation (*s*_*i*_) and/or competition from other species (negative *a*_*ij*_), enabling the community to reach an equilibrium state where the population densities of constituent species are static.

The gLV models have been criticized for their simplicity and some unrealistic properties. In particular, interaction terms capture the net effect of taxa upon one another, rather than having an underlying mechanistic basis. That is, they do not explicitly model features such as carbohydrate breakdown, or secretion of toxins. Moreover, interactions are unable to change over time or under different environmental conditions, while with strongly positive interactions, the basic equations predict unbounded upwards growth which is not reflective of real systems [14]. To prevent this unbounded growth, we include in our model an additional limit on total population sizes, analogous to population growth within a spatially limited environment. With this limit, each species will grow according to gLV growth dynamics until the total population size reaches a certain cap, after which point taxa will instead begin to compete, preventing the total population size from ever exceeding the cap. While this adaptation of the modelling assumptions does not qualitatively affect our results (S1 Fig), it both ensures against any unrealistic population explosions that can arise from the original gLV model and, importantly, allows for a coarse approximation of metabolic flexibility, with interactions able to change when the population reaches its cap.

While simple, the advantage of the gLV equations is that they can capture in high-throughput a wide range of complex ecological systems, including many of the constituent network motifs known to occur within microbial communities, such as networks of cross-feeding between species [15]. Due to this flexibility and breadth, the gLV equations are widely used to model both macroscopic and microscopic communities. And, importantly for our purposes, this use includes a growing number of examples that have applied the gLV model to microbiome data to generate testable, and subsequently supported, predictions [9,16–20].

**Fig 1:**
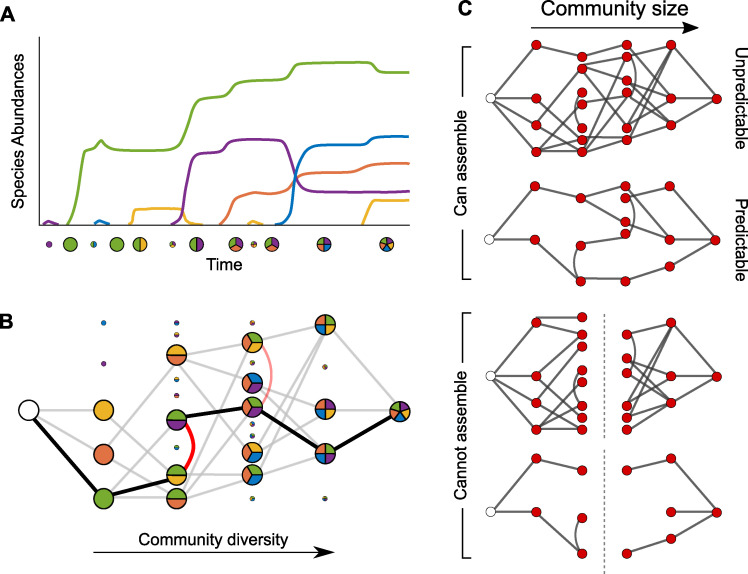
Mathematical models to simulate and map out community assembly. **(A)** One method of exploring community assembly is to draw species one by one at random from a large pool then assess whether and how they establish a diverse community. However, this approach only gives a single example of assembly per community, and due to the vast number of possibilities, we do not know whether other assembly pathways are possible. (**B)** In our alternative approach, we randomly select a diverse community, then determine which of its subcommunities are feasible and all possible transitions between them. In this manner, we can map out all the pathways by which a given community may have assembled (bold line in **B** indicates assembly pathway observed in **A**). (**C)** Examining the characteristics of a community’s assembly map enables us to determine whether it will be able to assemble from scratch and whether it will assemble in a predictable manner.

While the gLV model allows one to capture vast numbers of different communities and study their properties, the study of community assembly is a particularly challenging case. The reason being that, in addition to studying a final stable community, one is also interested in the many possible ways by which a given community might form. One intuitive way to proceed would be to randomly draw, in sequence, species from a large pool that represents all possible species and ask whether or not they are capable of forming a community of a given size [21,22] (Fig 1A). However, this approach suffers from the need to estimate the behavior of vast numbers of combinations of communities for each condition of interest. For example, from a pool of just 100 species, one can form over 17 trillion 10 species communities, each of which can in turn assemble by numerous different routes. Sampling all possible assembly pathways, for all possible communities within a given pool quickly becomes computationally intractable. As a result, comprehensively assessing the robustness or predictability of communities drawn from different pools becomes extremely challenging.

To overcome this problem, we devised a different approach, inspired by the study of adaptive landscapes comprised of sequences of beneficial mutations [23]. Here, a common approach is to take a well-adapted state—an individual with, say, 5 beneficial mutations—and then map out all of the potential ways to reach this adaptive state via different sequences of acquiring the 5 mutations. By analogy, we developed a method to build complete assembly maps for microbiome communities (Fig 1) [24–27]. Rather than attempt to build communities from the bottom up at random, the key to our method is to start with viable climax communities of a certain size. We then work backwards to systematically identify all the possible pathways comprised of smaller communities from which they could have assembled (Fig 1B). By doing this, for any given community, we can define a directed network that captures all the paths by which that community can assemble. By analyzing the properties of this network, we can then both determine whether the community is able to assemble and quantify assembly properties, such as the most likely state the community will be in, and whether it will assemble in a predictable manner (Fig 1C).

Overall, our approach is equivalent to observing a particular microbiome community in nature and trying to reconstruct artificially each of the paths by which it might have assembled. This method has proved a valuable tool for experimentally analyzing both micro- and macroscopic ecosystems [27], with experimentalists reconstructing all possible routes to assembly in artificial microcosms, or via retrospective data analyses. Such approaches have uncovered the importance of order-of-arrival effects in diverse contexts, including aquatic ecosystems [28,29] and small mammal communities [30]. Importantly, for our approach, this work has also demonstrated the ability of simple pairwise interactions to predict more complex community compositions [31].

By focusing only on viable final communities—and excluding those species and communities that fail to establish—the number of communities that need to be studied using these top-down approaches are vastly smaller than when working from the bottom up. A necessary limitation to this method is that it cannot capture species that only transiently colonize and are not present in the final community. However, our analyses suggest that typically such species are rare for our modelling assumptions (S1 Text). In this way, the problem becomes manageable and we are able to seek general rules of microbiome assembly.

### Strong interactions place limits on community assembly

The nature of species interactions is known to be critical to the properties of ecological communities. For example, communities with strong, cooperative interactions can be less stable than their less interdependent counterparts [32–34]. We begin by asking, therefore, whether the assembly of a given community is also affected by the strength and sign of the interactions between species. Using our approach, we can examine the assembly maps of communities with any combination of interaction frequency, type, and strength—enabling us to comprehensively analyze how interspecies interactions influence community assembly.

**Fig 2:**
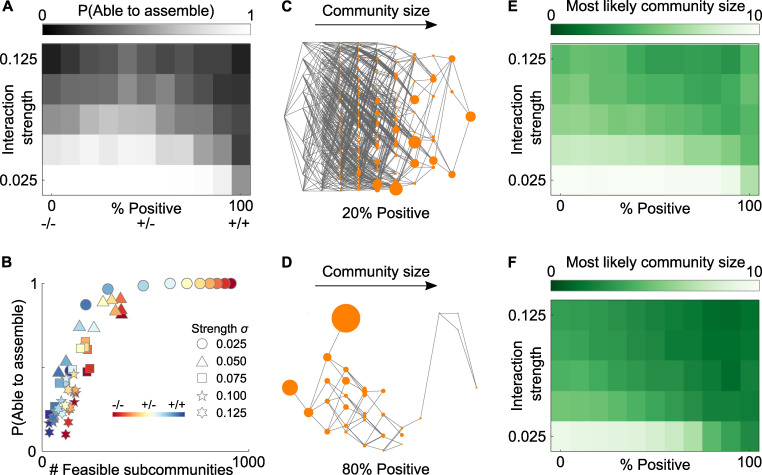
Strongly interdependent communities are harder to assemble. **(A)** Strong interactions (moving bottom to top) reduce the probability a community will be able to assemble via the random, sequential arrival of individual species. Symmetrical interactions (−/− and +/+) are also more of a barrier to assembly than asymmetrical ones, with the most robust assembly seen in the middle of the figure (high number of +/− interactions) moving left to right. (**B)** A reduced ability to assemble stems from low numbers of feasible subcommunities, which lead to splits in the assembly map, such that there is no single path from uncolonized to fully colonized (Fig 1C). (**C, D)** Treating the assembly map as a transition matrix allows us to calculate the probability of observing the community in a given state when subject to the continuous arrival and loss of taxa. In this illustration size of dot represents the probability of observing each subcommunity at any given time (here gamma / delta = 20). (**E, F)** Heatmaps illustrate the most likely community size for communities that are capable of assembling when (**E**) the rate at which species arrive is much greater than the rate at which they are lost ($\frac{\gamma }{\delta }$ = 20) or (**F**) when arrival and loss rates are similar ($\frac{\gamma }{\delta }$ = 2). In each case, even when communities are capable of assembling from scratch, they will not necessarily be able to in the face of continuous arrival and loss of taxa. The more interdependent, or more strongly interacting species are, the less diverse the microbiota is likely to be at any given time. For all analyses, climax communities are drawn with S = 10, connectivity C = 0.5, from 150 independent replicates, underlying data in S1 and S2 Data.

Mapping across parameter space, we find that communities with strong interactions are less likely to assemble than communities with more weakly interacting species (Fig 2A). Strong interactions are known to typically render communities unstable [32–34]. However, here we are focused only on established communities that are ecologically stable. It is not simply a lack of stability per se, therefore, that explains why a given community is difficult to assemble. Instead, communities with a lot of positive interactions, which render species dependent on one another, struggle to assemble due to a low number of viable subcommunities (Fig 2B). Even though these communities are stable once assembled, their intermediary stages of community development are often unfeasible. That is, the members of these smaller subcommunities cannot stably coexist. As such, there may be no continuous path from an uncolonized community to the fully developed system, and thus the community is unable to assemble from scratch via the sequential arrival of its constituent species (Fig 1C).

We also find that symmetrical interactions tend to disrupt community assembly (Fig 2A). In the case of competition (−/−), when species co-occur in a diverse community, they effectively keep one another in check—ensuring no one species is driven extinct by another and allowing for a stable climax community. However, in smaller subcommunities, this balance is lost, and species can easily drive one another extinct. Symmetrical positive interactions (cooperation, +/+) tend to be even worse for assembly. Strong beneficial interspecies interactions can mean that individuals bounce back quickly from perturbations in a diverse community. However, these same interactions mean species will struggle to grow in smaller subcommunities where their cooperative partners are missing. In the extreme case where each species relies on another to grow, the community cannot assemble from scratch because no individual species is capable of initially colonizing the environment. Together, these distinct mechanisms for negative and positive interactions render small subcommunities unviable, undermining the ability of stable climax communities to assemble from scratch.

We see, therefore, an important disconnect between the stability of a given community and its ability to assemble. This means some diverse communities may be relatively resilient to small perturbations, and yet unable to assemble species-by-species from a low diversity state, due to the fracturing of their assembly map (Fig 1C). In other words, certain communities that are stable to fluctuations in the species’ population size can nevertheless be prone to irreversible collapse should multiple species be lost at the same time, similar to “humpty dumpty” communities observed in macroscopic food-webs [22]. Indeed, such communities appear to occur in the human microbiome, with growing evidence that even transient doses of antibiotics can have long-term effects on microbiome diversity and composition, leading to the apparent presence of alternative steady states in composition within any one individual [4,35,36]. Our results show how prolonged periods of low diversity can result from interspecies dependencies, which limit the potential for the incremental recovery of a community after species loss. Wherever such “humpty-dumpty” communities occur, our results also suggest that other mechanisms must be at play to aid initial microbiota assembly. We discuss potential host mechanisms that may aid in assembly in the final section.

### Diverse cooperative communities may be rare, even when they can assemble

Our initial analysis asked simply whether a community, in principle, is able to assemble. However, this analysis does not give an indication of whether a community is likely to assemble. Therefore, we developed an additional method to predict the probabilities that different communities will assemble. This asks: What is the likely state of a given microbiota given the potential for continual arrival and loss of taxa? Our approach treats community assembly as a continuous-time Markov chain process, in which transitions between states are determined by the gain or loss of individual species, and these transitions are independent of past transition steps. Specifically, we assume that individual new species will arrive within a community at rate *γ*, and individual species will be lost at rate *δ* (we assume these gain or loss events occur rarely enough that only one such event will ever occur during any given time-step). With these rates, we can then use our assembly map to construct a generator matrix that describes the rate at which a given microbiota will transition between states. From this generator matrix, we can then calculate the stationary distribution of our Markov process, which in effect tells us the probability of observing each feasible subcommunity at any given time (Fig 2C and 2D).

Analyzing our assembly maps in this manner reveals a key observation: Even when communities are capable of assembling from scratch, they may still be unlikely to reach a diverse, fully assembled state. Instead, unless the pace of species arrival far outstrips the rate of species loss (*γ*>>*δ*), the most likely community state is one of lower diversity (Fig 2E and 2F). Mapping across parameter space, we find this is particularly true when communities have large numbers of positive interactions and resulting dependencies between species (Fig 2E and 2F). The more positively and the more strongly interacting species are, the less diverse the overall microbiota community is likely to be at any given time (Fig 2E and 2F). What drives this phenomenon? Communities where species depend on each other for growth tend to have far fewer upward paths within their assembly map—that is, there are fewer opportunities for new species to establish within a subcommunity at any given time. As a result, the realized rate at which new species establish within subcommunities is far lower when species interact positively than when they compete. In some cases, this means existing species will be lost more quickly than new species are gained, rendering it challenging for a diverse community to assemble. As a consequence, strong dependencies between species can introduce substantial barriers to community assembly, even when they do not prevent it entirely.

### Strong positive interactions increase predictability of assembly

**Fig 3:**
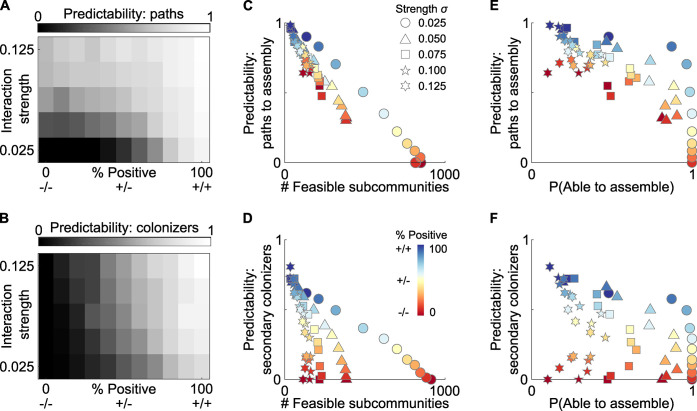
Strong, positive interactions increase the predictability of assembly. **(A)** Increasing interaction strength, and the number of positive interactions, decreases the number of edges within the assembly map. (**B)** Strong and positive interactions also increase the incidence of secondary colonizers, who cannot establish within the community until other species are present. Together, these factors increase the overall predictability of community assembly. (**C, D**) Increased predictability stems in part from a lower number of feasible subcommunities. (**E, F)** The relationship between interaction strength and sign and predictability leads to a trade-off between the robustness and predictability of assembly. For all analyses, climax communities are drawn with S = 10, connectivity C = 0.5, from 150 independent replicates, underlying data in S3 Data.

Our analysis reveals that communities with strongly positive interactions will struggle to assemble from scratch. This phenomenon has an important corollary: If such communities can assemble, they will do so more predictably. Specifically, the more species depend on one another, the fewer paths there are within the assembly map, and thus the fewer potential routes there are to community assembly (Fig 3A). Moreover, the more positive interactions between species, the higher the incidence of secondary colonizers—species that are unable to establish within the community until other species are present (Fig 3B). This predictability is driven in part by the smaller number of feasible subcommunities—the fewer feasible subcommunities there are, the fewer paths to assembly (Fig 3C). However, this is not the only mechanism at play—highly competitive communities also tend to have fewer feasible subcommunities but exhibit few secondary colonizers and thus less ordered assembly (Fig 3D). Why is this? As competitive species cannot benefit from the presence of others, they are, necessarily, more capable of growing on their own. Therefore, each species is capable of colonizing individually, and we do not see species that cannot establish until others are present. By contrast, the stronger the positive interactions within a community, the less capable species are of colonizing initially and the more limited are the routes by which a community may assemble, enforcing a predictable order to community assembly.

Importantly, while the most predictable assembly occurs for high levels of positive interactions, in which species are strictly cooperating with one another (each relies on the other to grow), we also see predictability when species are interacting through commensalism or exploitation (one species relies on another but not vice versa, Fig 3). This is important, as while bidirectional cooperative interactions may be relatively rare in the microbiome, strong one-way positive interactions between species appear to be more common and can occur whenever the waste products of one species are taken up and metabolized by another [37]. In both cases, our theory suggests these strong positive interactions are critical to the pattern of assembly, with the overall predictability of community assembly increasing as cooperative, exploitative, or commensal interactions increase (Fig 3A and 3B). However, this also raises a potential trade-off for a host: More predictable communities may be less likely to assemble at all (Fig 3E and 3F).

### Data from the human microbiota links ecological dependence and predictability

Our models lead to a number of predictions on microbiome community assembly. For a given community to robustly assemble, strong competitive or cooperative interactions between species must be rare. However, when strong positive interactions do occur, they are likely to play an important role in shaping microbiota predictability—enforcing an order to assembly, with species only able to colonize once their beneficial partners are present. Testing these predictions requires data on not only the assembly patterns but also the ecological interactions within a given community, which are challenging to obtain. One of the best-studied microbial communities is the human dental microbiota. Consistent with our predictions, this system has long been known for the predictable sequence by which species colonize, and there is experimental evidence of dependencies between a number of species whereby certain species rely on others for attachment to enamel and nutrients [38,39]. However, the dental data lack a quantitative assessment of the sign and strength of ecological interactions across the whole community, which is required to fully examine community assembly.

**Fig 4:**
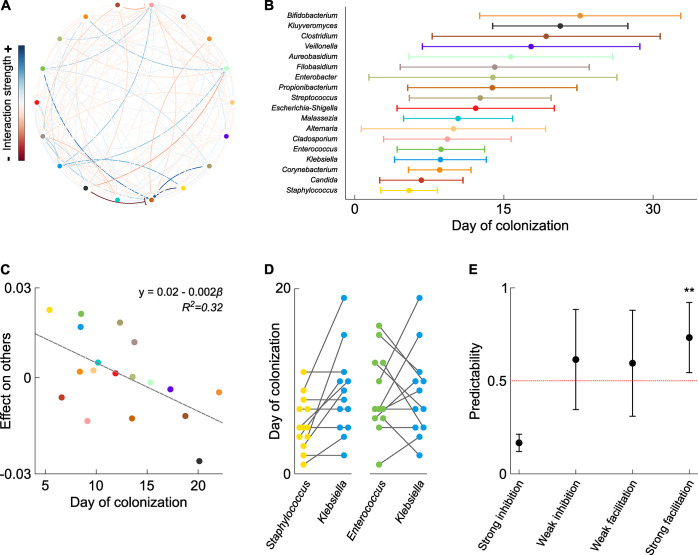
Published data reveal predictable assembly dynamics within the preterm infant gut, driven by interspecies dependencies. **(A)** Fitting data from 13 preterm infants sampled on a near daily basis for their first 6 weeks of life to the generalized Lotka–Volterra equations reveals a rich network of microbe–microbe interactions playing a strong and consistent role in shaping microbiome dynamics. (**B)** The preterm gut assembles in a robust and predictable manner, with specific taxa colonizing at different points in time. (**C)** More helpful taxa, determined by average effect on others, tend to colonize earlier in infant life (*R*^*2*^ *= 0*.*32*, *p = 0*.*02*). (**D)** We quantify the predictability of individual pairs of genera by calculating the proportion of infants in which the focal genus only colonizes with or after the partner genus. (**E)** Genus pairs in which the focal taxon receives a strong benefit from its partner (interaction *α_ij_* > 0.1) display predictable assembly dynamics, with the focal taxon significantly more likely to only colonize with or after its partner than expected by chance (average predictability score 0.73 > 0.5, one-sample permutation test *p* = 0.002). Underlying data in S4 Data.

To seek this information, we turned to a recently published analysis mapping microbiota assembly within the preterm infant microbiota [9] (Fig 4). The study combined high-frequency sampling of the preterm infant gut with ecological analyses to chart how the microbiota assembles and infer the sign and strength of the interactions between the constituent taxa. Specifically, a conservative regularization framework was used to fit the gLV equations to longitudinal data from 13 preterm infants, whose microbiotas were sampled near daily for their first 6 weeks of life. This approach identified a rich network of directed interactions occurring between genera within the infant gut that were predicted to play a strong and consistent role in shaping overall community dynamics (Fig 4A). A concern with these model fitting approaches is that they can overfit and infer interactions incorrectly. To address this, the authors then used a combination of mono- and co-culture experiments to empirically test each of the predicted interactions occurring between the 5 most prevalent taxa in the study [9]. Specifically, they grew each of these 5 taxa in isolation and in co-culture to assess the pairwise effect of each microbe on its partner’s growth in vitro and in a subset of cases, in vivo. Crucially, each of the interactions predicted to occur between these dominant taxa could be recapitulated experimentally, confirming that, though simple, the gLV fitting was able to accurately infer microbe–microbe interactions.

In line with previous work, this study found that the preterm gut assembles in a robust and predictable manner, with specific genera establishing at different points in time. Some genera typically colonized within the first week of life, while others typically did not establish until several weeks after birth (Fig 4B). In line with our theoretical predictions, strong cooperative or competitive interactions were rare, constituting only approximately 15% of all observed interactions. While rare, our theory suggests that strong positive interactions will nonetheless play an important role in shaping the predictability of assembly. Consistent with this, we find that genera that are, on average, more helpful tend to colonize earlier in infant life in the infant data (Fig 4C, *β* *= −0*.*002*, *R*^*2*^ *= 0*.*32*, *p = 0*.*02*). This pattern suggests that these beneficial species may be colonizing early and facilitating the arrival of others.

To look in more depth at the possible link between interaction type and the predictability of assembly, we turned to the individual colonization dynamics of each genus within the community, across each of the 13 infants. Specifically, for each focal genus, we calculated a Predictability score associated with each possible partner taxon. This score was defined as the proportion of infants in which the focal taxon only established after, or alongside the partner taxon, i.e., the extent to which the focal genus appears to depend upon the other genus to colonize. A score substantially greater than 0.5 implies that the focal taxon typically only establishes with or after its partner. A score less than 0.5 implies the focal taxon typically establishes before its partner, and a score around 0.5 implies the pair do not show any predictability in their colonization dynamics.

Calculating this score across each possible pair of taxa within our cohort revealed substantial variability in the predictability of assembly between taxa. Some pairs, such as *Klebsiella* and *Staphylococcus*, displayed highly predictable dynamics, with one genus never colonizing before its partner (Fig 4D, *Klebsiella-Staphylococcus* score = 1). Meanwhile, others such as *Klebsiella* and *Enterococcus* demonstrated no apparent predictability, with each genus equally likely to colonize first (Fig 4D, *Klebsiella-Enterococcus* score = 0.58). However, classing interactions based on their sign and strength, we found that focal genera receiving a strong benefit from their partner were significantly more likely to only colonize with or after that partner genus than one would expect by chance (Fig 4E, average predictability *score = 0*.*73 > 0*.*5*, one-sample permutation test *p = 0*.*002*). Furthermore, we found a positive correlation between the extent to which a focal genus benefited from its partner and its predictability. That is, the more a focal genus benefited from its partner, the more likely that focal genus was to consistently only colonize with or after that partner (Linear mixed effects model predictability ~ β*interaction_strength with genus as multimember random effect, *β = 0.76*, 95% *CI*: *0*.*04 to 1*.*40*). Quantifying predictability in the assembly process is challenging, and thus these analyses should be interpreted with caution. Nevertheless, these results are consistent with our prediction that strong, positive interactions between taxa are rare but can play a significant role in the predictability of community assembly.

### Host control of assembly

Our models suggest that that diverse communities can face significant barriers to assembly—particularly when species interact in a strong, positive manner, suggesting that even stable communities may be unable to assemble from scratch. Nonetheless, many host-associated microbiomes are composed of large numbers of species, and there is growing evidence of rich cross-feeding networks between species. For example, in the adult human gut, there appears to be widespread cross feeding of both polysaccharide breakdown products and short chain fatty acids, notably acetate, among bacterial species [15,37]. These metabolic dependencies are also important for the host, as they result in the production of compounds, including butyrate, which are taken up and metabolized by host cells [40,41]. These observations beg the question: How is it possible to reliably assemble communities with dependencies present that can undermine the process?

**Fig 5:**
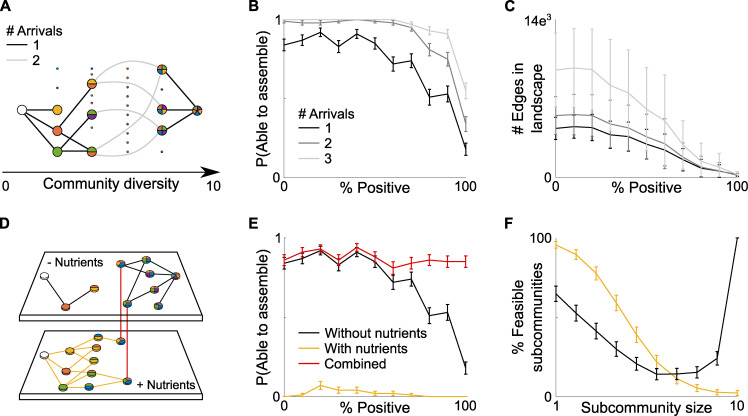
Multiple arrivals and host feeding can each increase a community’s ability to assemble. **(A)** Species arriving together rather than individually enables them to bypass the interspecies dependencies that would otherwise block community assembly. (**B)** These multiple arrivals increase the probability a community will be able to assemble. (**C)** However, multiple arrivals also increase the number of edges within the assembly map, reducing the predictability of assembly. (**D)** We capture variable host-feeding in our framework with a multilayer network composed of the assembly map for a community with and without supplemental nutrients provided by the host. If a subcommunity is feasible in both layers of the network, we assume that at that point in assembly, the host can add/remove nutrients without disrupting the current subcommunity. This enables continuous assembly pathways that span both network layers. **(E)** This host feeding can increase the probability of a community assembling, provided the feeding is removed once the community is sufficiently diverse. (**F)** It is beneficial for the host to provide additional nutrients early in assembly but not late, as these nutrients increase the feasibility of small subcommunities but reduce the feasibility of larger subcommunities. Here, for all results, climax communities are drawn with species number S = 10, connectivity C = 0.5, interaction strength sigma = 0.05, from 100 independent replicates, underlying data in S5 Data.

To address this, we sought to identify processes that can improve the assembly of interdependent species. One potential solution is to ensure that species colonize simultaneously. During natural birth, for example, human infants [42] have the potential to be inoculated with diverse multispecies communities from the mother’s microbiota. In addition, FMT studies have demonstrated the effectiveness of implanting entire communities into one [43]. To explore the role of such processes, we extended our model to allow consortia of multiple species to arrive together (Fig 5A). As expected, this does indeed allow communities to assemble that were previously unable to do so (Fig 5B). When species arrive together, they allow communities to jump gaps in their assembly maps and escape low-diversity dead-end communities. For communities that are already able to assemble from scratch, these multiple arrivals partially undermine the predictability of assembly, increasing the number of edges within the assembly map, and thus the number of paths by which a given community can assemble (Fig 5C). However, the key feature of predictability will remain: Species that can only colonize diverse communities will still be unable to establish within the host until others are present. As a result, species are expected to remain divided into the same pioneers and secondary colonizers. We also still see some communities that are unable to assemble from scratch—here species are so interdependent that almost every other member of the final community must be present for any individual species to persist. In these cases, unless the complete microbiome community is transplanted into a new environment together, the community will be unable to assemble from scratch.

We hypothesized that another candidate mechanism to overcome the problem of dependencies is to provide nutrients that reduce the reliance of symbionts on cross-feeding. For example, it is notable that human milk contains a wide range of oligosaccharides that can be consumed by certain members of the microbiota, but not the baby [44]. To explore such effects, we extend our model to capture the assembly of community under changing environmental conditions. Specifically, we consider a final environment with the climax community and an earlier environment that differs in that the community receives a growth boost due to supplemental nutrients. For any given climax community, we can now generate 2 distinct assembly maps—one defining assembly dynamics without any external input, and one defining how the community can assemble in the presence of host-provisioned nutrients (Fig 5D). Moreover, we identify any overlaps between these 2 maps; that is, subcommunities that are feasible both in the presence and absence of host-nutrients. These subcommunities act as potential transition points, at which a host could add or remove nutrients without disrupting the community as a whole. Together, this method enables us to form a connected multilayered network that describes assembly in these 2 different states, alongside the potential transitions between them that are under host control (Fig 5D).

With our method, we can now study the impact of host-provisioned nutrients on the ability of a community to assemble. Consistent with our initial hypothesis, we find that an early growth boost to the microbiota increases the probability that a given community will assemble from scratch (Fig 5E). Additional nutrients enable taxa to colonize that would otherwise be unable to, due to their overreliance upon other community members. Importantly, this result rests upon any feeding being reduced or stopped once a diverse community has been established (Fig 5E). This occurs because if a host’s climax community is already feasible and stable (nutrients are not needed to keep the system afloat), then increasing nutrient provision can cause more harm than good. Specifically, by promoting stronger competitors over weaker ones, feeding can lead to competitive exclusion and species extinctions, which prevent the community from reaching its climax state (Fig 5F). In sum, our model suggests that the key to using nutrients to aid assembly is to feed early, but then shut this off once species are in place.

Our theory suggests host-provisioned nutrients can facilitate species establishing early in assembly, but may impede microbiota development if they are not removed once communities are more complex. The importance of host-provisioned nutrients is supported by 2 studies that tracked the dynamics of infant microbiome assembly during early childhood [45,46]. As predicted by our theory, and consistent with previous work [47], the receipt of breastmilk appeared to facilitate the early colonization of certain members of the microbiome, with breastfeeding associated with higher levels of *Bifidobacterium*, a genus thought to play an important role in infant health [46]. Moreover, and again as predicted, both studies also found that the removal of breastmilk appeared to be just as important for infant microbiota development later in life. Specifically, the continued use of breastmilk later in microbiota development appeared to slow further microbiota assembly. Infants who still received breastmilk at 12 months remained dominated by *Bifidobacterium*, even after the introduction of solid food. Here, the microbiome remained more similar to that of younger infants than for individuals that did not receive milk, and the capacity to degrade certain polysaccharides did not manifest within the microbiota until breastmilk was removed. In this example, there is no suggestion that the prolonged use of milk is in anyway harmful. Nevertheless, these observations are consistent with our predictions that nutrient provision early in life may facilitate microbiota assembly but can slow it if continued down the line.

## Conclusions

Here we have developed ecological theory in order to investigate the rules governing the assembly of microbiome communities. We chose to use relatively simple ecological models because these allow one to study large number of communities and possibilities and seek general principles. As a consequence, there are a number of factors that have the potential to influence community assembly that we have not explicitly considered. Network structure, dynamic regulation of interactions, and metabolic plasticity [48,49] may each buffer against species loss and reduce the extent to which microbes depend upon one another, potentially playing a key role in aiding the assembly process. Disentangling the role these factors and others are likely to play in microbial community assembly is an important area for future research. Nevertheless, our analyses suggest that the general ecological properties of species—the manner in which microbes interact both with one another and their host—can play a central role in the ability of a diverse microbiota to robustly assemble. Strong, symmetric, and positive interactions between species may form barriers to community assembly, reducing the probability that a given community can assemble from scratch, even if it is stable once established. Such interactions lead to sparse assembly maps such that communities can get stuck at states of low diversity. However, when strongly interacting communities can assemble, we expect them to do so much more predictably, because of the relatively few viable routes by which assembly can proceed.

We also see that barriers to assembly can be avoided if a host can pick up consortia of species together, or provide assistance to community members, particularly during the early stages of community development. The uptake of multiple symbiont species in one step occurs in species where parents actively feed their fecal microbiota to offspring. This behavior is seen in animals as diverse as koalas [50] and termites [51], which both rely on the assembly of a specific, and metabolically complex, microbiota for nutrient acquisition. Mother–offspring symbiont transfer is more passive in humans but, as discussed above, there is growing evidence that host-supplied glycans, both in milk and as a major component of secreted mucins at the epithelium, are a major nutrient source for some members of the microbiota [52–55]. Indeed, it appears that humans may have evolved a wide repertoire of mechanisms, ranging from nutrient secretion to immunoglobulin A (IgA) production, to promote taxa that otherwise struggle to colonize the gut [56,57]. These processes may prove critical to the initial assembly of the microbiota as well as the potential to reassembe after perturbations such as antibiotic treatment [58]. Our work emphasizes the need to increase the understanding of the ecological interactions within microbiome communities. Only by taking into account the ecology of the microbiome will we be able to understand and ultimately manipulate the microbiome.

## Materials and methods

### Modelling microbiota dynamics

In order to properly capture the dynamics of microbiome assembly, we require an underlying community dynamics model that is not constrained by strict assumptions about community structure. To achieve this, we capture microbiome community dynamics using the gLV equations. Under this model, the rate of change of each species *i*, with a community of size *S*, is given by,

$$\frac{dX_{i}}{dt}=X_{i}\left(r_{i}-s_{i}X_{i}+\sum_{j=1,j\neq i}^{S}a_{ij}X_{j}\right)$$

where *r*_*i*_ represents the intrinsic species growth rate, *s*_*i*,_ captures the effect of a species upon itself (termed the self-regulation), and *a*_*ij*_ the effect of species *j* upon species *i*. These interspecies interactions (*a*) can take 1 of 5 possible forms based on the signs of *a*_*ij*_ and *a*_*ji*_; exploitative (+/−), competitive (−/−), cooperative (+/+), commensal (+/0), and amensal (−/0), and crucially, we can manipulate the proportions of each of these forms in order to fully examine how specific interaction types affect community assembly (S1 Text). In our case, communities are initiated as purely inhibitory, such that all interactions are either competitive or amensal. To examine the role of interaction types upon assembly, we then gradually increase the level of facilitation, *P*_*m*_, within the community by switching individual *a*_*ij*_ chosen at random to be positive, such that the proportion of exploitation and commensalism increases and at *P*_*m*_ *= 1* all interactions within the community are either commensal or cooperative. The magnitude of these interspecies-interactions are drawn from a half-normal distribution, with standard deviation *σ*, used to set the average strength of interspecies interactions.

Importantly, we adapt our implementation of the gLV equations with an additional population cap, analogous, for example, with there being a limited amount of space within the environment. Specifically, we assume that each species, *i*, will grow according to gLV growth dynamics until the total population size reaches a cap, *K*^*T*^, at which point species will instead begin to compete for space. To model this, once the community has reached the cap, at each time point, we first calculate species growth rates using the standard gLV model, then use these to calculate normalized a growth rates for each species. A species whose individual growth rate equals the average rate over all species will have a normalized growth rate of zero, species growing at above the average rate will have a positive normalized growth rate, and species growing at below the average will have a negative normalized growth rate. The effect of this is that once the total population has reached the environmental capacity slow growing species will begin to decrease in abundance as they are outcompeted by faster growing community members. Importantly, this cap not only prevents infinite growth but also introduces the potential for higher-order interactions, similar to those observed in individual based models of microbiota dynamics [34]. Specifically, at high population sizes species will begin to compete for the limited space within the environment, even when their intrinsic interactions are classified as cooperative (i.e., a_ij_ and a_ji_ are both positive). This higher-order competition will tend to stabilize highly cooperative communities that would otherwise be rendered unfeasible due to unbounded growth [34]. However, comparing our analyses to the standard Lotka–Volterra model reveals that this cap does not qualitatively affect our results (S1 Fig). Throughout, for our analyses, we set K^T^ = 10,000.

### Constructing assembly maps

As outlined in the main text, this work focuses on determining the assembly maps for given communities. To construct the assembly map for a given focal community, we first calculate all the possible subcommunities of a focal species pool that are capable of persisting within the environment (termed viable subcommunities). Viable communities are those in which each constituent species has a nonnegative density and is able to recover following perturbations to species densities—defined for simplicity here are being those that are linearly asymptotically stable. We then determine all the possible transitions between them via the arrival of a new species, introduced at a low density. These transitions fall into 3 key categories: (1) The new species forms a viable community with the existing species, increasing overall community diversity; (2) The new species replaces an original community member, maintaining current community diversity; or (3) The new species drives multiple existing members extinct, reducing overall community diversity. Initially, we assume that new species arrive rarely enough that we only need to consider the arrival of species one at a time; however, we later extend this to consider multiple arrivals. In this way, we can capture all possible developmental pathways of a habitat from initially uncolonized, through various subcommunities, to a final state in which no further species can be added (Fig 1).

### Incorporating host feeding

We incorporate host-feeding as an addition of a constant, *f*, to the intrinsic growth term, *r*_*i*_, in the gLV equation, such that species dynamics follow $\frac{dX_{i}}{dt}=X_{i}({(r}_{i}+f)-s_{i}X_{i}+\sum_{j=1,j\neq i}^{S}a_{ij}X_{j})$. We then construct 2 assembly maps, one in the presence of host feeding and one in the absence of feeding (*f = 0*), to create a multilayered network (Fig 3D). When subcommunities are feasible under both scenarios, we introduce an edge between the 2 network layers, indicating that no species within the subcommunity in question will be lost if feeding is removed/introduced. If the only continuous assembly paths possible span both network layers, then we conclude that the community can only assemble if the host provides additional nutrients for at least part of the assembly process.

### Analyzing clinical data

To test whether our predictions bear out, we analyze the interactions shaping microbiota dynamics in a published dataset tracking microbiota assembly within the preterm infant gut [9]. In this work, the authors collected microbiota samples on a near daily basis from 13 preterm infants residing in a Boston tertiary-care neonatal intensive care unit. Absolute abundances of different microbial taxa were determined using the MK-SpikeSeq sample sequencing protocol. Interspecies interactions were quantified by fitting these data to gLV models. Specifically, the authors used Bayesian spike-and-slab variable selection to fit the following to their longitudinal data,

$${\dot{X}}_{il}=\frac{\Delta \ln X_{i}(t_{l})}{\Delta t_{l}}=r_{i}+\sum_{j=1}^{M}\alpha_{ij}X_{j}(t_{l})+\sum_{k=1}^{N}\epsilon_{ik}E_{k}(t_{l})$$

where the left-hand side of the equation represented the logged change in abundance of genus *i* over time window *l*, *X*_*j*_*(t*_*l*_*)* represented the geometric mean of genus *j* across the time window *l*, and *ϵ_ik_* represented the effect of antibiotic *E*_*k*_ on genus *i*. This allowed the estimation of each genus’ intrinsic growth rate and interactions with community members. For the analyses in Fig 4, we define interactions whose normalized magnitude fell below 0.1 as weak, and interactions with a normalized magnitude greater than 0.1 as strong. We fit the linear mixed effects model using the MCMCglmm package in R [59], with *n = 10*,*000* iterations.

## Supporting information

S1 Fig Capped and noncapped models give qualitatively equivalent results. Here climax communities are drawn with S = 10, connectivity C = 0.5, from 150/100 independent replicates for the capped and uncapped models respectively (see Materials and methods for model description, underlying data in S1 and S2 Data).
(EPS)

S1 Text Supplementary methods and analyses. (DOCX)

S1 Data Raw data underlying S1 Fig, right panel. (MAT)

S2 Data Raw data underlying Fig 2. (MAT)

S3 Data Raw data underlying Fig 3. (MAT)

S4 Data Raw data underlying Fig 4. (XLSX)

S5 Data Raw data underlying Fig 5. (MAT)
