# Supplementary material for: Ecological rules for the assembly of microbiome communities
Source: PLoS Biol. 2021 Feb 19;19(2):e3001116. doi: 10.1371/journal.pbio.3001116 (PMC7946185; doi:10.1371/journal.pbio.3001116)
Supplement: S1 Text — (DOCX) [file pbio.3001116.s002.docx]

**Supplementary Information**

**S1: Constructing climax communities**

To construct the interaction matrices that characterize our climax communities, we implement the following procedure, where *S* represents the number of species within the climax community, *C* its connectivity, and *P_m_* the probability that any one species will increase the growth rate of another (level of facilitation):

1. We generate an *S x S* matrix *M* in which each entry is assigned a random variable drawn from a uniform distribution *M_ij_ ~ U(0,1)*.
2. We set those *M_ij_ <= C* to *M_ij_ = 1*, and those where *M_ij_ > C* to *M_ij_ = 0*. This generates a binary matrix determining which species within the community interact with one another, and in what direction (though not yet in what manner). The higher C, the more species will interact with one another.
3. We generate a second *S x S* matrix, *Q*, with entries again drawn from a uniform distribution *Q_ij_ ~ U(0,1).*
4. We set those *Q_ij_ <= P_m_* to *Q_ij_ = 1*, and those where *Q_ij_ > P_m_* to *Q_ij_ = -1*.
5. We then set This generates a matrix with entries [-1, 0, 1] that together describes which species interact with one another, and the nature of the interaction (i.e. positive or negative).

1. Finally, we generate an *S x S* matrix *N* with entries drawn from a half-normal distribution *N_ij_ ~ HalfNormal(σ)*. Our final interaction matrix is then given by,

1. For simplicity we assume each species has the same strength of self-regulation, so we set each

For this work, we are only interested in the assembly of communities that are feasible and stable once assembled. To enforce this, for any given climax community we draw its equilibrium densities from a uniform distribution such that each . We then set each species’s intrinsic growth rate, *r_i_*, based on the values necessary to maintain this equilibrium community. Specifically, we set,

Finally, we calculate the eigenvalues, of the Jacobian of this system when at equilibrium. If the maximum real part of these eigenvalues, max(Re())>0, then this climax community is unstable so we reject it and draw a new one. We repeat the process above until we have selected a stable community. It is important to note, the intrinsic instability of strongly interacting or cooperative communities mean more of these communities will be rejected than equivalent but more weakly interacting / competitive communities.

**S2: Constructing the assembly map**

A climax community of size S will have 2^S^ possible sub-communities, including the uncolonized state. Therefore, the assembly map can be described by a single 2^S^  x 2^S^ matrix, Q, in which each entry Q_ij_ represents the ability of sub-community *i* to transition to sub-community *j* via the arrival of one or more new species. In order to construct the assembly map for a given climax community we follow a series of steps outlined below, using the climax community T = [A B C D] as an example.

1. Calculate which sub-communities of T are viable, based on whether they have a feasible steady state (ie one where each of the species present has a non-negative density)
2. For each viable sub-community, identify each of the other sub-communities that could be reached via the arrival of a single new species - termed the augmented communities. For example, for sub-community i = [A B] these would be j = [A B C] and k = [A B D].
3. For those augmented communities that are also viable, add a connection in the assembly map, i.e., if *j* is also viable, set Q_ij_ = 1.
4. For those augmented communities that are not viable, calculate the growth rate of the new species when introduced to the initial community at a low density, i.e., calculate whether this species would be capable of invading. For example, in our case, if community k = [A B D] was not viable, check the growth rate of [D] when introduced to [A B]. If this growth rate is positive then numerically simulate the dynamics following the introduction of [D] until the community reaches a numerical steady state *l* (in most cases, during the simulations several species will have gone extinct).
5. If *l* is itself feasible and stable, we set Set Q_il_ = 1. If not, we determine the sub-subcommunities of *l* that are viable, *m*, and set Q_im_ = 1 (ie we assume the community will eventually drop into one of these following a subsequent perturbation).
6. Repeat this process for each of the viable sub-communities of T.

**S3: Transient colonizers in a constant environment are rare, fast growing, and helpful**

A key limitation to our assembly map approach is that, by definition, it cannot capture scenarios wherein species were present during the assembly process, but absent from the final climax community. To assess the role such species play in community assembly, we selected each of the communities in our main analysis that were unable to assemble and asked whether we could identify missing species that could facilitate assemble. Specifically, for each community we drew a further set of N=25 species that interacted randomly with the original community members and had a randomly selected intrinsic growth rate, *r,* yielding 7750 new species in total. We then assessed whether any of these species could enable the original community to assemble. Our analysis revealed that such species tend to be rare, with less than 1% of randomly drawn new species being capable of aiding community assembly. Intuitively, these ‘helper’ species tended to be faster growing than equivalent species that could not enable community assembly (mean *r^helper^* = 0.161 vs *r^unhelpful^* = 0.005, *tstat* = -3.66 , *p* = 0.0002). Notably though, even with these additional helper species, the overall trend in the ability of communities to assemble remained, with highly interdependent communities having a lower probability of being able to assemble than their more competitive counterparts.

Note that here we are considering communities assembling over time scales where the abiotic environment is relatively static, and thus dynamics are driven primarily by microbe-microbe interactions. For example, as observed for gut microbiota assembly during the first six weeks of life in preterm infants^9^. In practice, environmental shifts such as those following dietary alterations may lead to changes to intrinsic growth rates and to the nature of microbe-microbe interactions, in turn leading to the loss of species that had already colonized. In other words, environmental changes may generate the scenario where species that were central to the initial assembly process are absent from the final climax community. Such dynamics could be captured by within our modelling framework by combining multiple assembly maps designed to capture different environmental contexts, as enacted for our host-feeding model. Moreover, crucially, within each of these assembly maps, we expect our modelling predictions to hold.
